# The Medical Directory

**Published:** 1908-09-12

**Authors:** 


(*To the Editor of* The Hospital.)

### THE MEDICAL DIRECTORY.

(*To the Editor of* The Hospital.)

Sirs,—On the 1st inst. we posted to each member of the profession our Sixty-fifth Annual Circular. Will you kindly allow us, through your columns, to ask for its early return to us. If, by any chance, a practitioner has not received the form, we shall be glad to send one on application. It is particularly requested that the necessary alterations from Volunteer appointments to the Territorial Force should be accurately stated.

We are, Sirs, your obedient servants,
The Editors of the "Medical Directory."
7 Great Marlborough Street, London, W.

